# Meningitis as a recurrent manifestation of anti-AQP4/anti-MOG negative neuromyelitis optica spectrum disorder: a case report

**DOI:** 10.1186/s12883-021-02133-8

**Published:** 2021-03-09

**Authors:** Chenyang Zhang, Kang Zhang, Bing Chen, Jiao Yin, Miaomiao Dong, Yixin Qin, Xiao Yang

**Affiliations:** 1 Neuroscience Center, General Hospital of Ningxia Medical University, Key Laboratory of Craniocerebral Diseases of Ningxia Hui Autonomous Region, Yinchuan, 75004 China; 2 Department of Neurology, Beijing Tiantan Hospital, Capital Medical University, China National Clinical Research Center for Neurological Diseases, Beijing, China; 3 Department of Radiology, General Hospital of Ningxia Medical University, Yinchuan, 75004 China

**Keywords:** Neuromyelitis optica spectrum disorders, Anti-aquaporin-4, Anti-myelin oligodendrocyte glycoprotein, Meningitis, Intracranial infection

## Abstract

**Background:**

Neuromyelitis optica spectrum disorders (NMOSD), a group of autoimmune neurological diseases, involve the optic nerve, spinal cord, and brain. Meningitis is rarely reported as the primary clinical manifestation of both anti-aquaporin-4 (AQP4)/ anti-myelin oligodendrocyte glycoprotein (MOG) antibody-negative NMOSD (NMOSD^neg^).

**Case presentation:**

A 30-year-old man initially presented with fever, headache, and neck stiffness. Lumbar puncture revealed mixed cell reaction and decreased glucose levels. As a result, tuberculous meningitis was suspected. After 1 month, the patient developed longitudinally extensive transverse myelitis and area postrema syndrome. This was followed by the presentation of meningitis-like symptoms once again in the third attack, but his condition eventually improved after corticosteroid treatment without relapse for 2 years. However, he was readmitted to our hospital owing to symptoms of diplopia, hiccup, and numbness in the right hand. Brain magnetic resonance imaging (MRI) revealed that the area postrema still contained lesions. Spinal MRI revealed several segmental enhancements at the C4–C5, T1, and T5 levels. Anti-AQP4 and anti-MOG antibodies were persistently absent in the serum and cerebrospinal fluid (CSF). The patient was finally diagnosed with NMOSD^neg^.

**Conclusions:**

Meningitis could be a recurrent manifestation of NMOSD^neg^ and requires more careful evaluation.

## Background

Apart from being the primary manifestation of intracranial infection, acute meningitis also serves as an indicator of immune diseases. It is commonly observed in patients with autoimmune diseases, such as systemic lupus erythematosus (SLE) and Behcet’s disease [1]. Moreover, with the development of antibody detection technology, patients with meningitis/meningoencephalitis who were previously misdiagnosed with intracranial infection have been diagnosed with antibody-related immune meningitis/meningoencephalitis. For example, autoimmune glial fibrillary acidic protein astrocytopathy (A-GFAP-A), which was recently discovered, can also present with clinical symptoms mimicking infectious acute meningitis [2]. The discovery of these diseases has broadened the spectrum of immune meningitis/meningoencephalitis.

Neuromyelitis optica spectrum disorders (NMOSD), a group of autoimmune neurological diseases, involve the optic nerve, spinal cord, and brain. At present, it is more likely to be considered an autoimmune astrocytopathic disease, such as A-GFAP-A, rather than an inflammatory demyelinating disease, as previously reported [3]. The aquaporin-4 (AQP4) antibody, as a biomarker for diagnosis, is present in 80% of patients with NMOSD [4]. To date, only few cases of AQP4 antibody-seropositive NMOSD have been reported with acute meningitis/meningoencephalitis as the clinical manifestation. Furthermore, a subset of patients in the remaining patients with AQP4 antibody-seronegative NMOSD who presented with fever, headache, epilepsy, and imaging meningeal involvement were found to be positive for the myelin oligodendrocyte glycoprotein (MOG) antibody. MOG antibody-associated disease is now considered a disease group independent of NMOSD [3]. However, research on the clinical and imaging features of patients with NMOSD^neg^ is limited. Here we report the case of a patient with NMOSD who presented with fever, headache, neck stiffness, and double negative expression of the AQP4 and MOG antibodies so as to explore the diversity in the clinical presentations of NMOSD^neg^.

## Case presentation

A 30-year-old Asian man suddenly experienced symptoms of marked fever up to 40 °C, rigors, and serious headache in June 2015. After being admitted to local hospital, the initial suspicion was intracranial infection; however, anti-infection therapy had no effect. He was admitted to our neurology ward 1 month after symptom onset, with fever of 39 °C and neck stiffness; However, he was conscious. Peripheral blood count revealed a white blood cell (WBC) count of 12 × 10^9^/L and neutrophils accounted for 89.7% of cells. Lumbar puncture revealed a cerebrospinal fluid (CSF) opening pressure of 180 mmH_2_0, a WBC count of 205 × 10^6^/L (61% neutrophils and 32% lymphocytes), and a protein level of 0.67 g/L, with normal glucose and chloride levels. Brain contrast-enhanced MRI revealed no abnormal findings. An intracranial infection of unknown etiology was suspected and empiric therapy with ceftriaxone and ganciclovir was subsequently administered for 5 days; however, the patient’s condition did not improve. The results of the Mycobacterium tuberculosis test and CSF smear were both negative. Vancomycin was added to the anti-infection therapy for 1 week; however, there were still no positive results. Except for elevated C-reactive protein levels (CRP, 30 μg/L) and erythrocyte sedimentation rate (ESR, 49 mm/h), extensive serological and microbiological examinations of the CSF, including examination for the presence of bacterial culture, brucella, syphilis, human immunodeficiency virus, as well as the virus nucleic acid and antibodies of herpes simplex virus I/II, cytomegalovirus, rubella virus, and toxoplasma, were all unremarkable. Repeated lumbar punctures showed little changes, whereas the glucose level in the CSF decreased (1.9 mmol/L). The diagnosis of tuberculous meningitis was considered taking into account the mixed cell reaction and decreased glucose level in the CSF, combined with the poor effect of anti-infection therapy. Accordingly, empiric antituberculosis treatment was initiated, alongside dexamethasone (10 mg/day × 1 week). Following this treatment, the patient’s symptoms improved. He was then discharged and continued to take antituberculosis drugs.

**Fig. 1:**
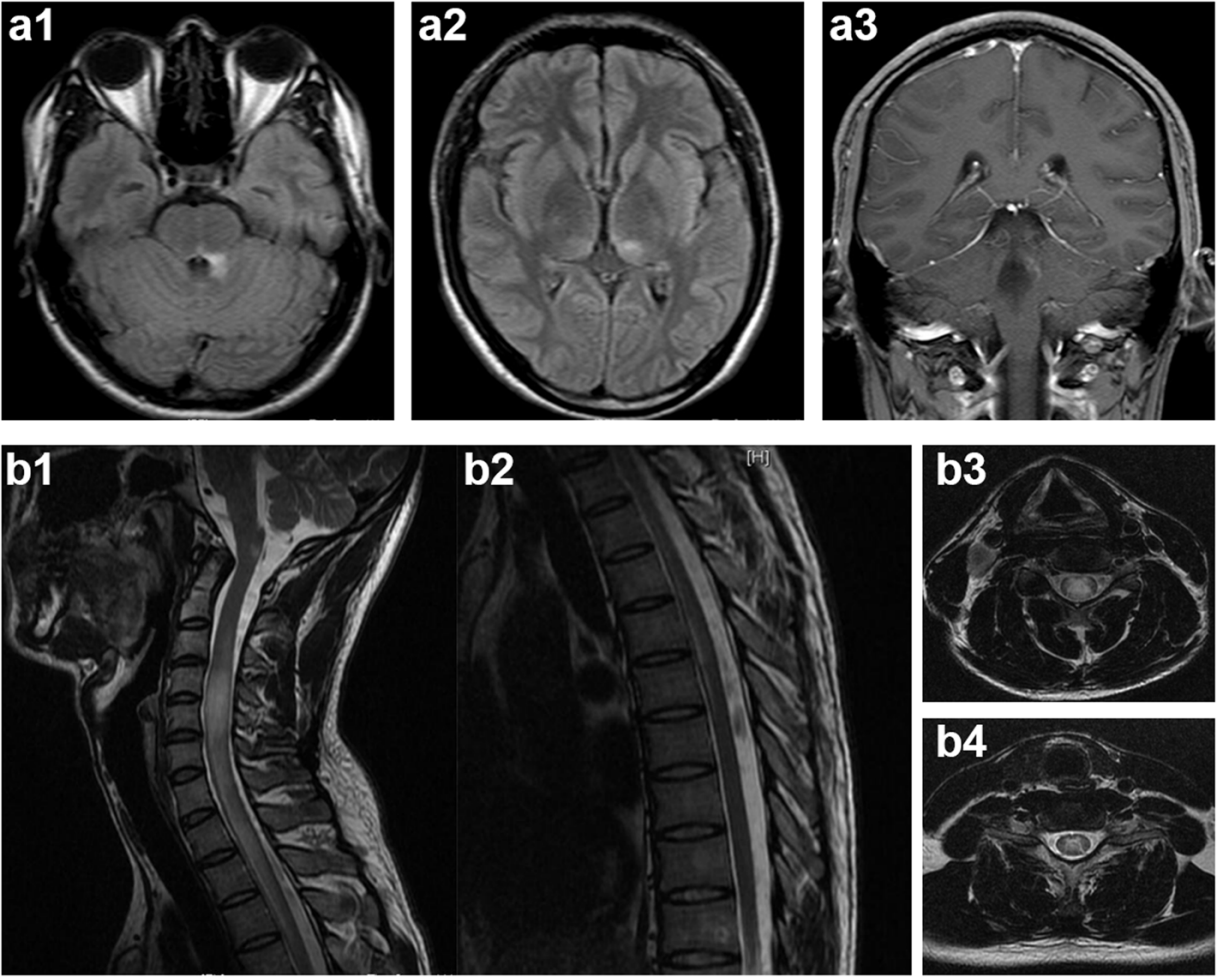
MRI findings of the patient during the second episode. a1, a2: Flake-like hyperintense signal lesions located in the dorsal pons and left medial thalamus in T2-FLAIR. a3: Enhancement in the dorsal pons. b1, b2: Lesions in the area postrema and C3–T6 levels of the spinal cord. b3, b4: Lesions were mainly located in the central gray matter of the spinal cord

The patient felt no discomfort; however, 1 month after his discharge, the symptoms of dizziness, numbness on the left side, and weakness in both lower limbs successively appeared. At this time, lumbar puncture results revealed no evidence of disease, but the patient was readmitted to our hospital in August 2015. Neurological examination revealed pain disorder below the chest and muscle strength of grade four out of five in both lower limbs. He also suffered from frequent hiccups during hospitalization. MRI revealed abnormal T2-weighted fluid-attenuated inversion recovery signals in the dorsal pons adjacent to the fourth ventricle, left medial thalamus (Fig. 1 a1-a3), dorsal medulla (area postrema), and gray matter of the spinal cord (C3–T6) (Fig. 1 b1-b4). Immune damage secondary to intracranial infection was subsequently considered; therefore, a thorough immunological examination was performed. The results showed that autoimmune antibodies tests both with a tissue-based assay and a cell-based assay in CSF and serum, including tests for anti-AQP4 and anti- N-methyl-D-aspartate receptor (NMDAR) antibodies, were all negative. The Oligoclonal bands and myelin basic protein in CSF were also negative. In addition, rheumatic-associated antibodies including anti-nuclear antibodies and anti-SSA/SSB antibodies, as well as the thyroid-related antibodies were all negative. Visual evoked potential was normal. Although all the immunological indicators were unremarkable, we still initiated high-dose methylprednisolone pulse treatment followed by an oral steroid with the dose tapered in 3 months, considering that all the pathogenic tests of the patients were negative, which eventually resulted in improvement in condition and subsequent discharge.

However, after 6 months, the patient experienced fever of up to 40 °C, rigors, and headache again in February 2016. He was then readmitted for the third time. Lumbar puncture revealed a WBC count of 22 × 10^6^/L (74% lymphocytes and 24% neutrophils), with normal protein, glucose, and chloride levels. Laboratory tests including blood count, CRP level, ESR, and T-SPOT, were normal. Brain MRI revealed that the previous brain lesions disappeared. Unfortunately, we did not perform enhanced MRI at this time. Furthermore, autoimmune tests for AQP4, MOG, and NMDAR antibodies were also negative and the diagnosis remained unclear. However, the patient’s condition markedly improved after receiving intravenous dexamethasone (20 mg/day for 1 week). He was discharged followed by no medication without relapse for 2 years.

Unfortunately, he experienced a fourth admission in April of 2018 with symptoms of diplopia, hiccup, and numbness in the right hand. He presented with subjective diplopia and brisk reflex in both lower limbs. Lumbar puncture revealed normal results. Brain MRI revealed that the lesions in the left pons tegmental area and area postrema had reappeared, similar to the second attack. Spinal MRI revealed several segmental enhancements at the C4–C5, T1, and T5 levels. Serum and CSF samples collected for reassessment were negative for AQP4, MOG, and GFAP antibodies. The patient was subsequently started on immunoglobulin pulse treatment, considering bilateral femoral head necrosis. Following this treatment, he entered remission and was subsequently discharged. Azathioprine was stopped because of elevated liver enzyme levels, and the patient refused to be treated with mycophenolate mofetil due to financial problems. The patient returned with no obvious symptoms in November 2018. Lumbar puncture revealed normal results. AQP4, MOG, and GFAP antibodies in the serum and CSF were all negative. The patient returned to normal life and work. Clinical follow-up has continued with no indication of relapse to date.

## Discussion and conclusions

The case in this study was characterized by recurrence and remission. There were a total of four clinical episodes, of which the second and the fourth were clinically and radiologically consistent with longitudinally extensive transverse myelitis (LETM) as well as area postrema syndrome, besides, corticosteroid treatment was effective. During the 5-year observation, we have excluded other diseases that could lead to similar impairment, such as systemic immune diseases, including SLE and Sjogren’s syndrome, considering that repeated systemic antibody tests were negative and the patient didn’t show multisystem involvement in the follow-up. Neuro-Behcet’s disease is observed more frequently in men than women and typically affects young adults aged 20 to 40 years. Since Neuro-Behcet’s disease can also involve brainstem, cerebrum, spinal cord or meninges, leading to encephalopathy, myelitis or meningitis, it becomes the first disease that came to our mind. However, oral aphthae, genital aphthae, skin lesions and ocular uveitis were all denied before the onset of the disease and during the five-year follow-up period, and several skin prick tests for the patient during hospitalization were also negative, all of which led us to rule out Neuro-Behcet’s disease. As a novel autoimmune astrocytic disease, A-GFAP-A has been widely reported to involve cerebrum, brainstem, spinal cord and meninges, and with meningoencephalitis as the main manifestation [5]. It is characterized by a relapsing-remitting course and responds well to glucocorticoids. Nevertheless, positive serum and CSF antibodies, especially CSF GFAP IgG, is the most important diagnostic basis of A-GFAP-A, and our patient’s serum and CSF GFAP IgG were both negative during the four episodes and subsequent follow-up, which makes the diagnosis of A-GFAP-A untenable. Then considering the clinical manifestations in addition to negative AQP4 antibody expression, the patient generally met the diagnostic criteria for NMOSD in 2015 [6]; therefore, he was diagnosed with NMOSD^neg^. However, the two episodes of meningitis-like symptoms during the course of this patient’s disease are rarely observed in NMOSD, which calls for our attention to distinguish them from intracranial infections in clinical settings.

The patient presented with fever, headache accompanied by neck stiffness, and elevated CSF WBC count during the first episode. He was therefore misdiagnosed with intracranial infection. Antibiotics were administered, although there was no etiological evidence. Subsequently, empirical antituberculosis treatment combined with short-term corticosteroid treatment, a common therapy in clinical practice, was initiated owing to noneffective antibiotic treatment, following which the patient’s condition rapidly improved. However, he developed LETM and area postrema syndrome after 1 month during the second episode. Therefore, how do we consider the patient’s first episode?

Viral infection could serve as the antecedent event in NMOSD attack. Levinson et al. reported the case of a patient with AQP4 antibody-seropositive NMOSD with Sjogren syndrome who was positive for Epstein–Barr virus (EBV) in the CSF during the acute phase. The author is inclined to consider the NMOSD attack secondary to the EBV infection [7]. To demonstrate the relationship between infection and NMOSD, Koga et al. performed serum pathogenic tests of 24 viral and bacterial infections in 19 patients with AQP4 antibody-seropositive NMOSD. The results showed that 7 of 15 patients had a higher number of infection-related antibodies in the acute phase; this number was significantly higher than the number in the control group. The researchers concluded that the early viral infections might be a trigger of subsequent NMOSD onset [8]. However, none of these patients had experienced meningitis-like symptoms, such as fever, headache, and neck stiffness. Recently, a study reported a case of NMOSD relapse where the patient primarily presented with meningoencephalomyelitis, headache, fever, and pleocytosis. Antibiotic therapy combined with corticosteroids was administered, which finally resulted in relative improvement. The author concluded that suspected bacterial meningoencephalomyelitis was the presentation or trigger of NMOSD attack in this case [9]. Reportedly, the CSF cell count is greater than 50 × 10^6^/L in 13–35% of patients with NMOSD [10]. Thus, whether or not our patient’s first episode including fever, headache, and pleocytosis was a preceding infection must be determined. The review of our case resulted in the finding that the diagnosis of tuberculous meningitis in the first episode was unreliable and was based on (1) the fact that there was no etiological evidence of tuberculosis infection throughout the course of the disease; (2) the fact that the patient’s symptoms of fever and headache rapidly improved within a few days after both antituberculosis and dexamethasone treatment; and (3) the fact that antituberculosis treatment lasted only 1 month, which is far from sufficient for the treatment of tuberculous meningitis. Furthermore, CSF findings completely returned to normal within a very short period of time (1 month), which is almost impossible in patients with tuberculous meningitis. These findings indicate that the patient’s first onset was not caused by tuberculosis or other pathogens and that the remission of symptoms was obviously associated with short-term corticosteroid treatment. Furthermore, the patient presented with meningitis-like symptoms again in the third episode and achieved relief with corticosteroids alone, which provides further evidence that the meningitis-like symptoms of fever and headache were the presentation of NMOSD attack and not that of an antecedent infection or trigger.

As an exceptionally rare phenotype of NMOSD, aseptic meningitis has been reported in only a few cases of AQP4 antibody-seropositive NMOSD, in which most of patients initially presented with aseptic meningitis, whereas others experienced meningitis several years after the diagnosis of NMOSD [9, 11–15]. However, these patients were almost accompanied by myelitis, optic neuritis, and encephalopathy at the same time when they presented with the symptoms of aseptic meningitis. Cases with simple aseptic meningitis as the main manifestation, like in our patient, are very rare. During the last few years, MOG antibodies have been detected in an increasing number of patients with AQP4 antibody-seronegative NMOSD. According to the data, the frequency of AQP4 antibody-seronegative patients usually varies between 12 and 24% in NMOSD [16]. Furthermore, the MOG antibody was detected in approximately one-third to one-fourth of patients with AQP4 antibody-seronegative NMOSD [17]. Compared with AQP4 antibody-seropositive NMOSD, MOG antibody-associated disease usually leads to lesions in the brain cortex, and the manifestations of meningoencephalitis or meningoencephalomyelitis resembling acute disseminated encephalomyelitis are more common in MOG antibody-associated disease [17, 18]. Nagabushana et al. reported the case of a patient with aseptic meningitis with MOG antibody seropositivity who presented with fever, headache, and neck stiffness. However, the patient also had lesions in her optic nerve, insula, and temporal lobes at the same time [19]. Only a small number of patients merely present with aseptic meningitis even in MOG antibody-associated disease. Suzuki et al. reported the case of a 55-year-old woman with no history of disease who presented with fever, headache, and neck stiffness. The MOG antibody was positive; her condition improved after immunotherapy. However, the symptoms of optic neuritis and myelitis appeared 50 days after remission [20]. Recently, Shi et al. reported the cases of two young men who were positive for the MOG antibody and initially presented with fever, headache, and neck stiffness; they developed optic neuritis 10 days and 1 month after onset, respectively [15]. According to the literatures, only one case of NMOSD^neg^ with meningitis-like symptoms has been reported [21]. The patient, a 20-year-old Japanese woman, was admitted to the hospital with fever, headache and vomiting. Slightly elevated WBC count and protein level were revealed, then she was initially diagnosed with aseptic meningitis. However, brain MRI soon revealed lesions in the hypothalamus and the tegmental part of pons, and long-segment lesions in the cervical and thoracic spinal cord were also found during the further examination. Therefore, the diagnosis was revised to NMOSD, although AQP4 and MOG antibodies were negative. The author concluded that it is the hypothalamic lesion that affected the thermoregulatory center and resulted in fever rather than meningitis. In the four clinical episodes of our case, both the first and third attacks were merely characterized by meningitis, but there was no hypothalamus involvement throughout the course of the disease, combined with the presentations of headache, neck stiffness, and significantly elevated CSF WBC count, we still consider that the patient had aseptic meningitis. AQP4 and MOG antibodies detected in the serum and CSF three times during the ictal and interictal periods were negative. The patient has been followed-up for 5 years and no relapse has been seen in the past 2 years. At present, he has returned to normal life and resumed to work. No significant differences in clinical presentation, recurrence rate, or outcome between AQP4 antibody-seronegative and seropositive patients have been reported. It remains to be seen whether AQP4 antibody-seronegative NMOSD currently encompasses any heterogeneous diseases and whether a new NMO-associated autoantibody is present. To date, to the best of our knowledge, there are no representative studies that have reported about a patient with NMOSD^neg^ merely presenting with meningitis. However, we still believe that more attention should be given to such kind of patients, because negative antibody is more likely to lead to diagnostic confusion and missed diagnosis in clinical practice, which is very important for timely treatment and improving prognosis. The patient in our case will continue to be followed-up.

In conclusion, our patient demonstrates that meningitis could serve as a recurrent manifestation of NMOSD^neg^, which needs careful differentiation from intracranial infection.

## Data Availability

All data are included in the manuscript. There are no additional data.

## Abbreviations

A-GFAP-A: Autoimmune glial fibrillary acidic protein astrocytopathy
AQP4: Aquaporin-4
CSF: Cerebrospinal fluid
CRP: C-reactive protein
ESR: Erythrocyte sedimentation rate
EBV: Epstein–Barr virus
GFAP: Glial fibrillary acidic protein
LETM: Longitudinally extensive transverse myelitis
MOG: Myelin oligodendrocyte glycoprotein
MRI: Magnetic resonance imaging
NMOSD: Neuromyelitis potica spectrum disorders
NMOSD^neg^: Anti-AQP4/anti-MOG antibody-negative NMOSD
NMDAR: N-methyl-D-aspartate receptor
SLE: Systemic Lupus Erythematosus
WBC: White blood cell
